# Challenges in the measurement and valuation of oncological treatments in Spain: recommendations and call to action

**DOI:** 10.1007/s12094-026-04323-7

**Published:** 2026-05-06

**Authors:** Maria Rosario García-Campelo, Jesús Corral, Fernando Moreno, Andrés Munoz, Jesús M. Balea, Begoña Barragán, Jordi Ginés, Fernando Gutiérrez Nicolás, Bruno Sangro, Eva Martín Martin-Sánchez, Marta Trapero-Bertran

**Affiliations:** 1 Department of Medical Oncology, Complejo Hospitalario Universitario A Coruña (CHUAC), University Hospital A Coruña, A Coruña, Spain; 2 Hospital Universitario Virgen del Rocio, Seville, Spain; 3 Medical Oncology Department, Hospital Clínico San Carlos, Madrid, Spain; 4 Medical Oncology Department, Hospital General Universitario Gregorio Maranon, Universidad Complutense, Madrid, Spain; 5 Servicio Gallego de Salud, Santiago de Compostela, Spain; 6 Grupo Español de Pacientes Con Cáncer, Madrid, Spain; 7 Servicio de Farmacia, Hospital Universitario Son Espases, Baleares, Palma, Spain; 8 Department of Pharmacy, University Hospital of Canary Islands, Santa Cruz de Tenerife, Spain; 9 Liver Unit, Clinica Universidad de Navarra, Pamplona, Spain; 10 Early Assets Oncology, AstraZeneca, Madrid, Spain; 11 Applied Economics area, Economics and Business Department, Faculty of Law, Economics and Tourism, Universitat de Lleida, Lleida, Spain

**Keywords:** Uncertainty, Clinical endpoints, Access to oncology drugs, Spain, Sustainability, Strategic planning

## Abstract

**Background:**

The evaluation of innovative oncology medicines presents significant challenges related to the selection of appropriate clinical endpoints and the sufficiency of evidence, particularly in therapeutic areas, such as immunotherapies, targeted treatments, single-arm trials, and tumor-agnostic therapies. In these contexts, the added value of new treatments is often not adequately captured by traditional clinical trial endpoints, such as overall survival (OS), which remains the gold standard in oncology assessment. This article aims to analyze the main sources of uncertainty in the value assessment of oncology therapies in Spain, with a focus on the limitations of current endpoints and evidence-generation processes, and to provide recommendations for enhancing the recognition and assessment of additional clinical benefit.

**Methods:**

A multidisciplinary expert panel composed of twelve professionals, including medical oncologists, hospital pharmacists, health economists, and patient representatives, was convened to identify and discuss key sources of uncertainty in the value assessment of oncology treatments, particularly those related to clinical endpoint selection and evidence generation. The panel participated in three structured plenary sessions. Additional external experts were engaged to provide complementary input in areas, such as tumor-specific characteristics and statistical methodology. Consensus statements were developed through an iterative process of discussion, critical appraisal, and refinement across and between sessions.

**Results:**

The expert panel issued twelve recommendations to improve value assessment in oncology. These include tailoring clinical endpoints to treatment type, tumor characteristics, and stage; complementing overall survival with milestone analysis and quality-of-life measures; and standardizing real-world evidence collection across the healthcare system. The panel advocated for a national portfolio of prioritized endpoints, appropriate statistical methods by context, and the conditional use of early-phase data for decision-making. Additional recommendations addressed the use of synthetic control arms, flexible reimbursement models, advanced analytics (e.g., AI and Big Data), evaluator expertise, and the promotion of stakeholder training and transparency.

**Conclusions:**

Addressing the challenges of clinical endpoint selection and evidence generation is essential to reduce uncertainty in the value assessment of innovative oncology treatments. The twelve expert recommendations outlined in this study provide a structured roadmap to improve methodological consistency, enhance the relevance and robustness of clinical and real-world data, and promote a more adaptive and transparent evaluation framework. These proposals aim to support more evidence-based, equitable, and sustainable decision-making within the Spanish healthcare system, while aligning with broader European initiatives in oncology drug assessment.

## Introduction

Cancer remains one of the leading causes of morbidity and mortality in Spain, representing a substantial and growing socioeconomic burden [1–4]. In recent years, oncology clinical research has accelerated markedly, leading to the introduction of innovative diagnostic tools and targeted therapies that enable earlier diagnosis and more personalized treatment strategies aimed at improving clinical outcomes [5, 6]. This scientific progress has resulted in two major epidemiological trends: an increase in the absolute number of diagnosed cases, driven by demographic changes, such as population growth, aging, increased life expectancy, greater exposure to risk factors, and expanded screening programs; and, concurrently, a significant improvement in patient survival accompanied by declining mortality rates [2–4]. These improvements are largely attributable to advances in prevention, early detection, and the development and availability of new therapeutic approaches.

The Spanish regulatory and public reimbursement framework involves two main public bodies: the Spanish Agency for Medicines and Healthcare Products (AEMPS), which evaluates the clinical efficacy and safety of new medicines for marketing authorization; and the Interministerial Commission on Drug Prices (CIPM), which is responsible for public reimbursement decisions based on cost-effectiveness and budget impact. Once a medicine is approved and reimbursed at the national level, its provision is funded by the Spanish National Health System (SNS). However, because healthcare responsibilities are decentralized to the Autonomous Communities, regional disparities in access may persist. While decisions made by AEMPS and CIPM establish the overarching framework, regional authorities may further modulate access through complementary mechanisms.

While oncology clinical trial activity continues to expand, innovative therapies often encounter regulatory and evidence-related bottlenecks, including limited availability of robust data, which may delay patient access [7, 8]. Such delays are not merely administrative concerns; they have measurable consequences in terms of lost life years (LYs), and, reduced patient outcomes [9]. This situation underscores the need to implement streamlined, evidence-informed evaluation and adoption processes for oncology therapies [10, 11].

One of the most critical components of oncology value assessment is the selection of clinical endpoints to evaluate therapeutic efficacy and inform reimbursement decisions. Overall survival (OS) has traditionally been regarded as the gold standard due to its objectivity, reproducibility, and clinical relevance [6, 12]. However, important limitations of OS, including long follow-up periods, large sample sizes requirements, and potential confounding, due to subsequent therapies, have become increasingly evident, in modern trial settings [6–13]. Moreover, in early-stage cancers or multimodal treatment contexts, attributing gains in OS to a specific intervention can be challenging [12–14].

Importantly, OS does not fully capture the multidimensional nature of therapeutic benefit, especially in advanced-stage cancers where achieving incremental survival gains is inherently challenging [6, 14]. Novel therapies, such as targeted agents and immunotherapies, often produce delayed or sustained effects, necessitating trial designs and endpoints tailored to their specific mechanisms of action [14, 15]. This has led to growing interest in alternative endpoints, such as progression-free survival (PFS), event-free survival (EFS), and patient-reported outcomes (PROs), which may provide earlier signals of efficacy and enhance the clinical and patient relevance of trial data [6, 13].

Despite their promise, these alternative endpoints introduce additional uncertainty. Methodological heterogeneity, lack of standardization, and limited validation against hard outcomes, such as OS constrain their generalizability and complicate their incorporation into health economic models [6, 16]. Since cost-effectiveness analyses and budget impact assessments rely heavily on robust clinical data, uncertainties surrounding endpoint validity may undermine confidence in these evaluations and ultimately affect reimbursement decisions [5, 10].

These challenges are particularly pronounced in contexts involving single-arm trials, tumor-agnostic indications, and rare cancer populations, where conventional trial designs are often infeasible [17–20]. In such settings, atypical response patterns and the absence of comparator arms necessitate reliance on advanced statistical methods, surrogate markers, and real-world evidence to support regulatory and economic decision-making [8, 21]. However, these methodologies carry their own limitations and assumptions, which may further contribute to uncertainty.

The integration of clinical and economic uncertainty represents a major challenge for health technology assessment (HTA) bodies such as AEMPS or CIPM, as well as for regional authorities, who must balance the imperative of fostering therapeutic innovation with the need to ensure equity, efficiency, and financial sustainability within a publicly funded healthcare system [5, 10]. While these issues have been explored in various international settings, there is a lack of systematic, context-specific analyses focused on the Spanish oncology landscape [7]. This article aims to analyze the main sources of uncertainty in the value assessment of oncology therapies in Spain, with a focus on the limitations of current endpoints and evidence-generation processes, and to propose recommendations to improve the recognition and assessment of additional clinical benefit.

## Methods

### Expert recruitment and composition

A multidisciplinary expert panel was convened to explore the limitations of current clinical endpoints in oncology and to develop consensus-based recommendations for improving their use in health technology assessment (HTA) in Spain. Experts were selected through purposive sampling to ensure diversity in professional background, regional representation, and gender. All procedures complied with applicable ethical and data protection regulations. This process did not follow a formal Delphi methodology, but rather a structured expert panel approach combining individual interviews and iterative plenary discussions to develop the final consensus statements.

Experts were identified through purposive sampling based on their recognized experience in oncology care, health technology assessment, hospital pharmacy, health economics, patient advocacy, or healthcare management. Selection aimed to ensure diversity in professional background, institutional setting, geographic representation, and stakeholder perspective, in line with current reporting recommendations for consensus studies.

The final panel comprised eleven experts, including five medical doctors (four medical oncologists and one internist with extensive experience in oncology), three hospital pharmacists, one health economist, one hospital manager, and one patient representative. Among the participating medical oncologists, the main tumor areas of clinical focus included lung, breast, gastrointestinal, and hepatobiliary cancers. This composition reflects the range of key stakeholders involved in the evaluation, access, and use of innovative oncology therapies within the Spanish healthcare system.

### Process and interactions

A preliminary literature review was conducted to identify therapeutic areas in oncology where overall survival (OS) might fail to fully capture the clinical benefit of new treatments. The review highlighted three settings of interest: targeted therapies, immuno-oncology, and early-stage disease. These findings informed the design of semi-structured discussion guides used in the first round of expert consultations to identify the limitations of current endpoints.

A series of in-depth, one-hour interviews were then conducted with all panel members (four medical oncologists, one internist, three hospital pharmacists, one health economist, one hospital manager, and one patient representative) via Microsoft Teams to further explore these limitations. The objective was to explore additional areas of complexity in oncology drug evaluation and clinical benefit limitations. Based on this round, two additional scenarios were identified as particularly challenging: single-arm trials and tumor-agnostic indications.

Subsequently, three plenary sessions were conducted to consolidate the ideas gathered from the individual interviews. The first session, held in person, focused on the clinical and methodological limitations of current endpoints in immuno-oncology and targeted therapies. The second session, conducted online, addressed the challenges associated with innovative trial designs, specifically single-arm trials and tumor-agnostic indications. The third session, also virtual, was dedicated to a structured consensus-building process aimed at formulating a set of practical, stakeholder-informed recommendations to improve the recognition and assessment of additional clinical benefit. All panelists received briefing materials in advance and were encouraged to actively contribute to all discussions. Due to newly assumed professional responsibilities, the health manager was unable to participate from the third session onwards. Experts were asked to assess potential alternative endpoints and modifiers, such as tumor type, disease stage, mechanism of action, and prior lines of treatment, that may influence the interpretation and value attribution of oncology therapies. The selection of clinical variables was refined through targeted follow-up consultations, leveraging each panelist’s specific expertise by tumor type or context. The responses were then synthesized, validated, and integrated into a consensus framework. To ensure a common level of understanding, the briefing materials included concise explanations of the main statistical methods and endpoints discussed, and these concepts were further clarified during the plenary sessions with the support of experts in health economics and statistical methodology.

The final set of consensus statements was developed through an iterative process, combining expert opinion and evidence-based reasoning. All panelists reviewed and approved the final version.

## Results

Findings on the limitations of current endpoints and evidence-generation processes are presented across two analytical dimensions: (a) Therapeutic context, including targeted therapies, immuno-oncology, single-arm trials, and tumor-agnostic indications; and (b) Disease stage, distinguishing between early-stage and advanced-stage cancer settings.

## Therapeutic context

### Limitations of overall survival (OS)

While overall survival (OS) remains the gold standard in oncology, panelists identified significant limitations. These include the need for long-term follow-up to obtain mature data, reduced informativeness in indolent or slowly progressing diseases, risk of confounding from subsequent therapies, and difficulty demonstrating incremental benefits in diseases with already prolonged survival. Importantly, the validity of OS, and other conventional endpoints, must be contextualized according to the specific clinical scenario.

### Immunotherapy and targeted therapies

Immunotherapy and targeted therapies pose unique challenges for evaluation due to their distinct mechanisms of action. Unlike traditional cytotoxic treatments, these therapies often produce delayed yet durable responses, where initial progression may not indicate treatment failure. Atypical response patterns, such as pseudo-progression, hyper-progression, and dissociated responses, further complicate the interpretation of conventional endpoints like median OS and progression-free survival (PFS).

These characteristics underscore the inadequacy of standard metrics in capturing the full therapeutic benefit. As a result, panelists emphasized the need for adapted methodologies and novel endpoints to assess efficacy more comprehensively. Existing frameworks, such as the ESMO-Magnitude of Clinical Benefit Scale (ESMO-MCBS) and iRECIST, attempt to address these issues but are primarily applied retrospectively and remain underutilized in prospective clinical trials.

Several alternative approaches were discussed: (a) Milestone analysis, which evaluates survival at pre-specified time points and accommodates delayed treatment effects; (b) Time-restricted mean survival, which focuses on survival within defined intervals, excluding long-term outliers and improving interpretability; and, (c) Biomarker-based endpoints, such as minimal residual disease (MRD) via circulating tumor DNA (ctDNA), and criteria like “any tumor regression,” which may capture benefit in patients with stable disease under conventional RECIST 1.1 standards.

Despite their promise, these novel endpoints require further validation to ensure their clinical and regulatory relevance.

In the context of targeted therapies, median OS, PFS, and event-free survival (EFS) are informative for quantifying early benefit. However, the emergence of resistance crossing survival curves challenge the proportional hazards assumption, limiting the applicability of traditional statistical methods. In such cases, milestone analyses provide a more accurate assessment. Overall, experts agreed that broader adoption of novel endpoints depends on the availability of robust evidence and the development of methodological standards.

### Single-arm and basket trials

The evaluation of clinical benefit in single-arm and basket trials presents considerable methodological and interpretive challenges, particularly for tumor-agnostic indications. Key concerns identified by panelists include: (a) The lack of control groups, which introduces uncertainty in estimating clinical benefit and limits comparative assessment; (b) Heterogeneity of tumor types, with variable prognoses and treatment responses—especially seen with immunotherapy—complicating endpoint selection and result interpretation; and, (c) Absence of predefined efficacy criteria, especially given the lack of European or national guidelines tailored to tumor-agnostic treatments.

Although randomized controlled trials (RCTs) remain the gold standard, they are often infeasible in these settings. Alternative methods, including synthetic control arms and advanced predictive modeling, have been proposed to infer comparative effectiveness, though methodological concerns regarding validity and comparability persist.

Basket trials typically use overall response rate (ORR) and duration of response (DoR) as primary endpoints. However, their limited ability to capture survival benefit has been flagged as a barrier to funding and access. Emerging metrics, such as time to subsequent treatment, second PFS (PFS-2), and the Growth Modulation Index, offer promising alternatives but require further empirical validation. Milestone analysis was again highlighted as a valuable complementary tool.

The panel also stressed the importance of integrating real-world evidence (RWE) to complement trial data, particularly through conditional authorization mechanisms. Nevertheless, successful implementation of RWE strategies requires investment in digital infrastructure, data standardization, and increased awareness among clinicians.

## Disease stage

### Early-stage disease

In early-stage and potentially curative settings, panelists highlighted major challenges in using OS as a primary endpoint. Notably, over 80% of clinical in early-stage cancer rely on alternative primary endpoints due to ethical and logistical barriers in obtaining mature OS data. In diseases such as early breast or testicular cancer, long-term survival renders OS impractical and delays access to effective therapies.

Key alternative endpoints include: (a) Pathological complete response (pCR), which is strongly associated with long-term disease-free survival in certain aggressive cancers; (b) Minimal residual disease (MRD), particularly ctDNA, as an early indicator of treatment response and recurrence risk; and, (c) Quality of life (QoL) measures, which are especially relevant in early-stage settings where treatment-related toxicity must be balanced against potential cure.

Experts emphasized the need for systematic inclusion of these variables in evaluation frameworks. In Spain, however, the incorporation of QoL outcomes remains in an early stage of implementation.

### Advanced-stage disease

In metastatic or advanced-stage cancer, evaluation methods have had to evolve in response to increasingly prolonged survival. Several challenges were identified: (a) Delayed OS data acquisition, especially in slowly progressing diseases with multiple treatment lines; (b) Tumor heterogeneity, which can lead to site-specific progression not captured by aggregate response criteria; and, (c) Post-progression survival, where extended life expectancy blunts the observable impact of new treatments on OS.

Given these constraints, alternative endpoints, such as QoL, symptom control, and patient-reported outcomes, are gaining prominence. In palliative contexts, patients often prioritize these outcomes over traditional survival metrics.

Specific tumor types, such as metastatic breast and colorectal cancers, illustrate the need for tailored approaches given their biological heterogeneity, multiple treatment lines, and prolonged post-progression survival. Although abundant data exist on prognostic variables, the interpretation and application of such data in regulatory and reimbursement decisions remain challenging and require further methodological refinement [8, 12].

#### Consensus statements

Additionally, a summary of the recommendations for enhancing the recognition and assessment of additional clinical benefit is provided. Following the analysis of limitations, the expert panel agreed upon twelve consensus statements. These statements aim to guide improvements in evaluating the additional clinical benefit of innovative oncological treatments, particularly in the context of immunotherapy, targeted therapies, single-arm trials, and tumor-agnostic indications, spanning both early and advanced stages of disease.

#### Structure and Purpose

The consensus statements are organized to: (a) Support clinical endpoint selection and measurement (CS1, CS5); (b) Recommend alternative analytical approaches (CS2, CS6); (c) Incorporate patient perspectives in trials (CS3); (d) Promote the systematic use of real-world evidence (CS4, CS8); (e) Inform trial design and value-based assessment (CS7, CS9); (f) Encourage the adoption of new technologies (CS11); and, (g) Strengthen multi-stakeholder training and engagement (CS10, CS12). See Table 1 for a summary of the consensus statements.

**Table 1:** Consensus statements (CS1–CS12)

CS1	Clinical endpoint selection and measurement must consider the type of treatment (therapeutic regimen, intention, line of treatment, and mechanism of action), tumor type and subtype, and disease stage, incorporating the patient’s biopsychosocial perspective
CS2	In clinical scenarios where, median OS is insufficient to demonstrate the additional clinical benefit of a treatment, alternative methods (e.g., milestone analysis) and the joint use of complementary endpoints are recommended
CS3	Quality of life, toxicity, and patient-reported outcomes should be systematically included as key co-variables in clinical trials, adapted to the clinical scenario
CS4	Real-world evidence (RWE) should be collected, analyzed, and integrated using a standardized, structured, and consistent format, under a coordinated national approach, to reduce uncertainty around new treatments
CS5	A consensual and regularly updated portfolio of key clinical endpoints by cancer (sub)type, stage, and treatment mechanism should be developed, aligned with European standards, and accompanied by methodological guidance for evaluating incremental benefit
CS6	The most appropriate statistical methods for inference and estimation should be selected based on the clinical context and level of uncertainty, considering the strengths and limitations of available methodologies
CS7	In basket trials, single-arm trials, and other designs with high uncertainty, robust evidence from phase I or II trials may be sufficient to support authorization and reimbursement decisions
CS8	The use of synthetic control arms derived from structured RWE should be promoted to address the absence of comparators and complement evidence from single-arm trials
CS9	Flexible reimbursement models, including risk-sharing agreements based on real-world outcomes, should be implemented to manage uncertainty and allow ongoing reassessment of treatment value
CS10	Clinical professionals involved in medicine evaluation should receive specialized training and have experience in the relevant pathology. Transparency and alignment among stakeholders are essential
CS11	The adoption of emerging technologies, such as Big Data, AI, machine learning, and digital tools, should be encouraged to enhance trial design, RWE analysis, and synthetic control development
CS12	Awareness and training initiatives should target all stakeholders, including healthcare professionals and patients, to strengthen participation in medicine evaluation processes and foster shared understanding of value in oncology

## Therapeutic context

### Endpoint selection aligned with treatment and disease characteristics (CS1, CS2, CS5)

Clinical endpoint selection should reflect the therapeutic intention, tumor subtype, mechanism of action, and disease stage, while integrating a biopsychosocial perspective centered on the patient. OS remains a cornerstone but is limited in contexts, such as slowly progressing diseases, early-stage cancers, and therapies with novel mechanisms of action. Alternative endpoints, including milestone OS, pCR, EFS, and ctDNA, should be considered when mature OS data are unavailable or insufficiently informative.

**Fig. 1:**
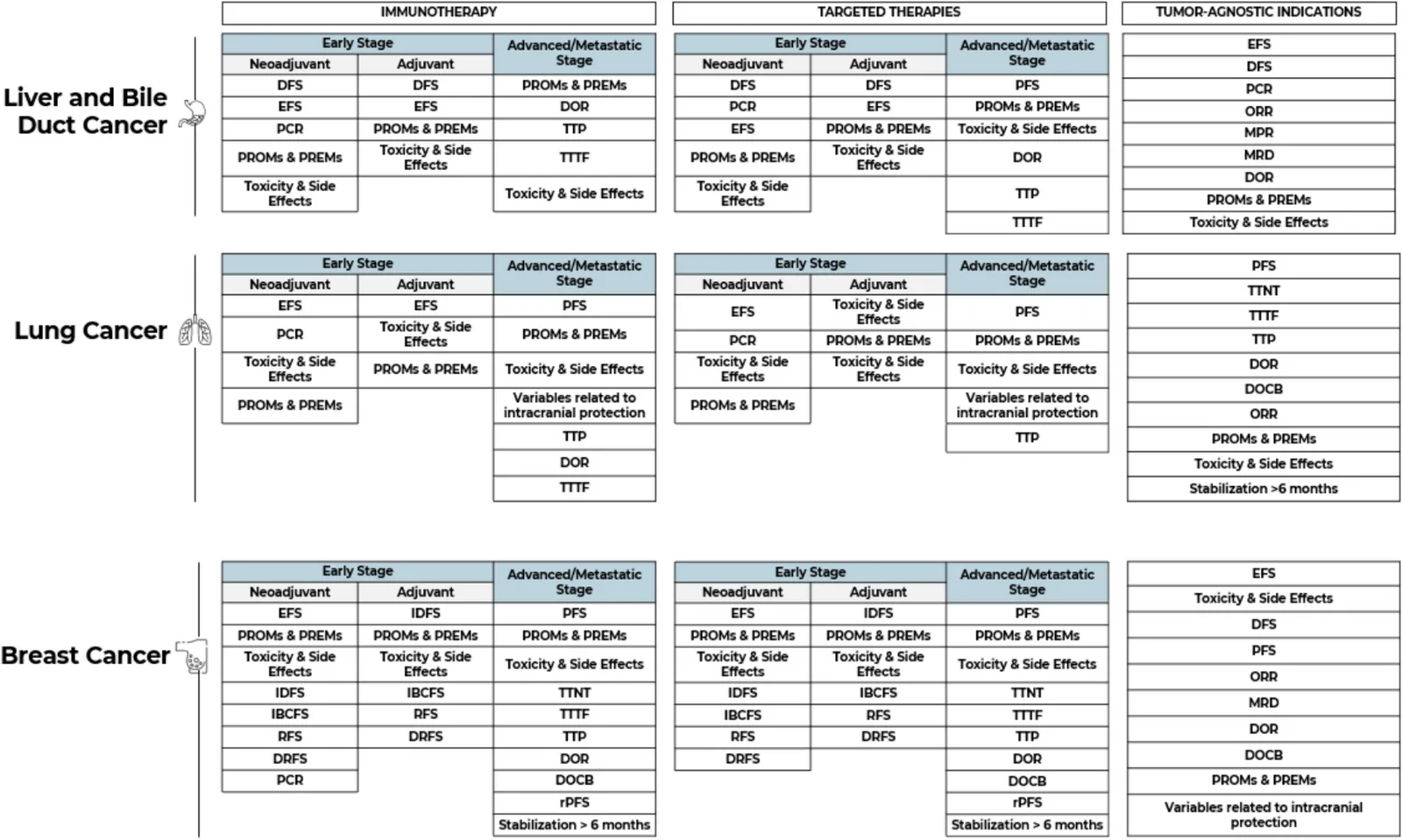
Recommended variables to consider when mature OS data are unavailable at the time of decision-making and/or to complement OS data. Summary of clinical scenarios

Panelists emphasized that each scenario (e.g., immunotherapy in NSCLC, targeted therapy in TNBC) requires endpoints adapted to the biological behavior and expected response patterns. In tumor types, such as hepatobiliary, lung, and breast cancer, recommendations varied by stage and subtype, suggesting specific variables (see Fig. 1) when OS is not practical or mature. Furthermore, a national, consensus-based portfolio of validated endpoints by disease context and treatment type (CS5) was recommended to ensure harmonization, equity in access, and guidance in HTA processes. The tumor types presented in Fig. 1 are illustrative examples derived from the areas of expertise represented in the panel and from the clinical scenarios identified in the preliminary literature review, and should not be interpreted as an exhaustive or prioritized list.

### Novel analytical strategies (CS2, CS6)

Where median OS does not reflect the full benefit of a treatment, statistical methods, such as milestone analysis, restricted mean survival time (RMST), and time-dependent covariate models, should be considered. These are especially suited to therapies with delayed but durable effects (e.g., immunotherapy) or settings where survival curves cross.

Advanced models (e.g., hierarchical Bayesian models, stratified Cox models, competing risks models, spline-based flexible models) are required in single-arm or basket trials, or when extrapolating beyond available data. Biostatistical expertise should be integrated early into trial design to align methodologies with expected response profiles.

### Clinical trial design and evidence generation (CS7)

In settings where RCTs are unfeasible, such as rare mutations or tumor-agnostic indications, evidence from well-designed phase I/II trials may suffice for regulatory and reimbursement decision-making. Panelists highlighted the importance of robust trial design, predefined endpoints, and context-specific statistical justification.

### Real-world evidence and synthetic controls (CS4, CS8)

RWE should be systematically collected and used to complement clinical trial data, especially where control arms are absent (e.g., single-arm trials). Synthetic control arms, constructed from structured and standardized RWE, can help contextualize treatment effects and reduce uncertainty. Ensuring data quality, standardization, and security is essential.

### Value assessment and financing models (CS9)

To manage uncertainty and ensure sustainability, the adoption of flexible reimbursement models linked to real-world outcomes is needed. These include performance-based agreements and conditional reimbursement contracts. Such models must be transparent, with clear mechanisms for data collection, reassessment, and renegotiation.

### Technological innovation (CS11)

Artificial intelligence, machine learning, and other data science tools should be leveraged to enhance endpoint selection, identify predictive biomarkers, analyze RWE, and design adaptive trials. These tools can also support digital twin modeling and anomaly detection in datasets.

## Disease stage

### Early-stage disease

In early-stage cancers, mature OS data are often unavailable due to long survival and curative intent. Thus, endpoints like pCR, DFS, IDFS, and ctDNA-based MRD are key to evaluating benefit. The panelists recommended using milestone analyses and predictive modeling to shorten timelines for decision-making without compromising robustness.

In breast and lung cancers, variable recommendations apply by subtype (e.g., TNBC, HER2 +, HR +) and treatment line (e.g., neo-adjuvant vs. metastatic), reflecting the heterogeneity in disease trajectories and recurrence patterns.

### Advanced-stage disease

In metastatic settings, prolonged survival and multiple treatment lines dilute the attributable impact of any single therapy. Traditional OS measurements often fail to capture nuanced treatment effects, especially when post-progression survival is long. QoL metrics, symptom control, and patient-reported outcomes (PROs) are therefore essential.

Incorporating these outcomes ensures that evaluations reflect real patient priorities, particularly in palliative care, and support a more holistic benefit assessment.

## Cross-cutting recommendations

### Patient-centered evaluation (CS3)

Inclusion of QoL, toxicity, PROs, and PREMs as co-primary or key secondary endpoints was strongly supported. The biopsychosocial perspective ensures the multidimensional impact of therapies is captured. Tools, such as Q-TWiST, SISAQOL guidelines, and disease-specific validated instruments, should guide this integration.

Shared decision-making and alignment with patient values are essential, particularly in advanced or incurable settings.

### Training, capacity building, and transparency (CS10, CS12)

Experts agreed on the need to train evaluators, clinicians, and patients in clinical endpoint interpretation, data analysis, and therapeutic innovation. Capacity-building initiatives should include joint training between clinicians and patients to develop a shared understanding of value.

Transparency in evaluation processes and inclusion of diverse perspectives, particularly from patients and regional systems, is essential to achieving equitable, patient-centered care.

## Discussion

Through successive discussion panels, a multidisciplinary group of experts developed twelve consensus statements and provided in-depth insights into the selection of clinical trial endpoints and optimal measurement strategies across diverse clinical contexts.

The evaluation of oncology medicines must be grounded in context-specific endpoints. As highlighted in consensus statements CS1 and CS2, while overall survival (OS) remains the gold standard, various clinical scenarios necessitate alternative approaches to adequately capture the therapeutic value of novel treatments [6, 14].

There was a broad consensus that the intrinsic value of individual endpoints varies depending on tumor type, disease stage, and treatment modality. Accordingly, endpoints should not be interpreted in isolation, nor treated as absolute measures. Instead, they should be jointly evaluated to reflect the multidimensional impact of oncological therapies.

Although OS remains highly relevant across all cancer types and stages, particularly in contexts where survival constitutes a major unmet need, its utility varies significantly depending on the clinical setting. Five limitations were noted: (i) OS can significantly delay patient access to innovative treatments due to the time required to achieve mature data, particularly in early-stage diseases, such as neo-adjuvant and adjuvant settings; (ii) Sole reliance on OS may obscure other clinically meaningful outcomes, including symptom control, quality of life, and progression-free survival (PFS); (iii) OS requires large sample sizes, limiting its feasibility in rare tumors or tumor-agnostic indications; (iv) OS is highly susceptible to confounding, especially in advanced or metastatic stages and first-line settings, where crossover or post-progression treatment changes may dilute its interpretability; and, (v) In the context of immunotherapy, traditional OS analyses may not adequately reflect therapeutic benefit due to delayed separation of survival curves and the importance of long-term survivors [13, 15].

In these contexts, alternative endpoints, such as milestone survival, restricted mean survival time (RMST), and patient-reported outcomes, offer more sensitive and context-appropriate measures (CS2, CS3) [13, 22, 23]. RMST is particularly useful when survival curves cross or when traditional statistical tests (e.g., log-rank) fail to detect clinically relevant differences [23]. However, the application of these methods requires advanced statistical support and expertise.

For tumor-agnostic treatments, the use of OS is further limited due to the low prevalence of relevant genetic alterations and the extended follow-up periods needed to demonstrate efficacy in the absence of control groups.

Given these challenges, the panel recommends the systematic inclusion of quality-of-life endpoints, PROMs, PREMs, and variables related to toxicity and side effects in all clinical trials (CS3), to ensure a comprehensive evaluation of treatment impact on patient experience.

In single-arm trials, the lack of a comparator arm necessitates alternative strategies for outcome assessment [17, 18]. The use of synthetic control arms and real-world evidence (RWE) (CS4, CS8) was highlighted as an essential complement to trial data [8, 21]. Incorporating historical controls and matched real-world datasets provides a comparative framework for evaluating clinical benefit.

Tumor-agnostic therapies introduce additional complexity due to heterogeneous response patterns across tumor types [8, 19]. Although overall response rate (ORR) and duration of response (DoR) are commonly used in European assessments, their limitations are well recognized [6, 16]. Alternative endpoints, such as Growth Modulation Index and time-to-next-treatment, offer further perspectives [17, 18], but require further validation before widespread adoption.

In early-stage disease, reliance on OS is particularly problematic due to the extended timelines required for data maturity and potential confounding from subsequent treatment lines [12, 13]. Surrogate markers, such as pathological complete response (pCR) and minimal residual disease (MRD),particularly through circulating tumour DNA (ctDNA), have emerged as early indicators of treatment efficacy and may correlate with long-term outcomes in specific clinical contexts [6, 14].

Reimbursement decisions must increasingly adopt flexible approaches to accommodate uncertainty in clinical evidence. Value-based payment models and risk-sharing agreements (CS9) are emerging solutions [5, 10], though their success depends on robust infrastructure and strong stakeholder collaboration [7, 24]. The decentralized nature of the Spanish healthcare system presents additional implementation challenges [5], requiring tailored strategies to address regional disparities.

RWE (CS4, CS8) has become indispensable for complementing clinical trial data [21, 25]. The analysis underscores the importance of standardized data protocols and interoperability across healthcare systems to ensure data quality and comparability [26]. International experiences have demonstrated the feasibility of implementing comprehensive RWE frameworks, although their adaptation to the Spanish context requires alignment with local healthcare structures and regulatory capacities [8]. Regulatory authorities should provide clear guidance on the systematic evaluation of non-randomised evidence, including criteria for endpoint selection and evidentiary thresholds [25].

Moving forward, several actions are necessary. A consensus-based portfolio of endpoints and accompanying methodological guidelines should be developed, drawing from international standards (e.g., ASCO, ESMO). Risk-sharing mechanisms should be rigorously assessed within the Spanish context. Continued investment in digital infrastructure and training is critical to sustain implementation efforts.

Implementation considerations (CS10, CS12) go beyond methodological aspects and include the need for specialized training and capacity building among professionals involved in evaluation. The increasing complexity of therapies calls for in-depth expertise in disease-specific mechanisms and evaluation methodologies [8, 16]. Initiatives such as Project Orbis offer valuable examples of harmonised, and accelerated regulatory evaluation [24].

The integration of emerging technologies (CS11), including artificial intelligence, machine learning, and digital tools, represents a promising direction for improving endpoint selection, RWE analysis, and the development of synthetic control arms [21, 26].

Several structural challenges remain. Clinical uncertainty, stemming from novel endpoints, innovative trial designs, and atypical response patterns, complicates interpretation of incremental benefit. Economic uncertainty, particularly in tumor-agnostic contexts, and a fragmented evaluation ecosystem, with divergent interpretations among stakeholders, further hinder consistent decision-making. Moreover, the lack of transparency and expertise gaps at regulatory and pricing levels represent significant barriers to the implementation of consistent and high-quality evaluation processes.

Overcoming these challenges requires a coordinated, collaborative effort among all relevant stakeholders, including regulatory agencies, clinicians, payers, patients, and researchers. The recommendations arising from this initiative are closely aligned with Spain’s Pharmaceutical Industry Strategy 2024–2028 [24], which advocates for reversible financing agreements, improved data collection, enhanced transparency, and the development of robust methodological guidelines for assessing additional clinical benefit and economic impact. The upcoming Royal Decree regulating the evaluation of health technologies is also expected to reflect many of these principles.

The main limitation of this study is the limited number of participating panelists, which may have constrained the ability to reach consensus on certain issues, particularly in specific clinical contexts. Additionally, although the hospital manager participated in the initial stages of the process, including the individual interviews and the first plenary session, they were unable to attend the final consensus meeting. However, the multidisciplinary composition of the remaining panel was maintained, and the consensus process continued with representation from the main stakeholder groups.

## Conclusions

The evaluation and adoption of new oncology medicines in Spain requires a coordinated, multidisciplinary effort to address the complex challenges outlined in this study. Through a series of expert panel discussions, several key areas for improvement were identified that could significantly enhance assessment processes and accelerate patient access to innovative therapies.

Recommendations include adapting evaluation frameworks to better accommodate non-traditional trial designs and endpoints, especially for immunotherapies, targeted therapies, and tumor-agnostic treatments. Their implementation will depend on sustained investment in digital infrastructure, continuous professional training, and active engagement from all stakeholders.

The findings underscore the importance of tailoring evaluations to the specific clinical and therapeutic context while maintaining scientific rigor. A more systematic use of real-world evidence, patient-reported outcomes, and advanced analytical methods may enhance both the relevance and robustness of assessment processes.

These recommendations may serve as a starting point for broader analyses and collaborative efforts aimed at advancing toward a more efficient, transparent, and equitable system for evaluating innovative oncology medicines, while safeguarding the sustainability of the healthcare system.

## Data Availability

The data supporting the findings of this study are based on expert panel discussions and do not include individual-level patient data. No datasets were generated or analysed. Additional information may be available from the corresponding author upon reasonable request.
